# Association of gene coding variation and resting metabolic rate in a multi-ethnic sample of children and adults

**DOI:** 10.1186/s40608-017-0145-5

**Published:** 2017-04-05

**Authors:** Jacklyn N. Hellwege, Digna R. Velez Edwards, Sari Acra, Kong Chen, Maciej S. Buchowski, Todd L. Edwards

**Affiliations:** 1 Division of Epidemiology, Department of Medicine, Vanderbilt Genetics Institute, Vanderbilt University Medical Center, Nashville, TN 37203 USA; 2 Department of Obstetrics and Gynecology, Vanderbilt Genetics Institute, Vanderbilt University Medical Center, 2525 West End Avenue, Suite 600, Nashville, TN USA; 3 Department of Pediatrics, Vanderbilt University Medical Center, Nashville, TN USA; 4 Diabetes, Endocrinology and Obesity Branch, National Institute of Diabetes and Digestive and Kidney Diseases, National Institutes of Health, Bethesda, MD USA; 5 Division of Gastroenterology, Hepatology and Nutrition, Department of Medicine, Vanderbilt University Medical Center, Nashville, TN USA

**Keywords:** Resting metabolic rate, Genetic variants, Gene-based analysis, Predicted gene expression, Obesity

## Abstract

**Background:**

Resting metabolic rates (RMR) vary across individuals. Understanding the determinants of RMR could provide biological insight into obesity and its metabolic consequences such as type 2 diabetes and cardiovascular diseases.

**Methods:**

The present study measured RMR using reference standard indirect calorimetry and evaluated genetic variations from an exome array in a sample of children and adults (*N* = 262) predominantly of African and European ancestry with a wide range of ages (10 – 67 years old) and body mass indices (BMI; 16.9 – 56.3 kg/m^2^ for adults, 15.1 – 40.6 kg/m2 for children).

**Results:**

Single variant analysis for RMR identified suggestive loci on chromosomes 15 (rs74010762, *TRPM1*, *p*-value = 2.7 × 10−6), 1 (rs2358728 and rs2358729, *SH3D21*, *p*-values < 5.8x10−5), 17 (AX-82990792, *DHX33*, 5.5 × 10−5) and 5 (rs115795863 and rs35433829, *C5orf33* and *RANBP3L*, *p*-values < 8.2 × 10−5). To evaluate the effect of low frequency variations with RMR, we performed gene-based association tests. Our most significant locus was *SH3D21* (*p*-value 2.01 × 10−4), which also contained suggestive results from single-variant analyses. A further investigation of all variants within the reported genes for all obesity-related loci from the GWAS catalog found nominal evidence for association of body mass index (BMI- kg/m^2^)-associated loci with RMR, with the most significant *p*-value at rs35433754 (*TNKS*, *p*-value = 0.0017).

**Conclusions:**

These nominal associations were robust to adjustment for BMI. The most significant variants were also evaluated using phenome-wide association to evaluate pleiotropy, and genetically predicted gene expression using the summary statistics implicated loci related to in obesity and body composition. These results merit further examination in larger cohorts of children and adults.

**Electronic supplementary material:**

The online version of this article (doi:10.1186/s40608-017-0145-5) contains supplementary material, which is available to authorized users.

## Background

Resting metabolic rates (RMR) in humans represent the energy required to sustain body functions under resting conditions, and varies across individuals [1]. Generally, RMR is lower in women than in men, and in older adults compared to younger adults, and is mainly dependent on the amount of muscle mass (and other metabolic tissues) [2]. Other variables impacting RMR include sleep duration [3], physical activity [4], and obesity [1, 5, 6]. As the amount of metabolic body tissue is strongly related to RMR, RMR per total body mass is reduced in obese individuals compared to normal weight individuals, due to their larger proportion of fat mass relative to the total body mass [2]. Moreover, RMR slows in response to weight loss in a phenomenon termed metabolic adaptation that acts to counter weight loss and is thought to contribute to weight regain [7].

Several studies have demonstrated the heritability of RMR (h^2^ ≈ 0.30) [1, 8, 9]. It has also been shown that RMR is lower in African than in European Americans, even in childhood [3, 10–13]. Some of this difference has been attributed to smaller organ size in African Americans [14] and reduced cardiorespiratory fitness [4]. Due to the recent rapid increase in the prevalence of obesity, especially in African Americans, a better understanding of genetic causes of inter-individual and racial variations in RMR is important to increase our knowledge of the biologic pathways contributing to obesity [15].

Although energy balance is critical to the development of obesity, few studies have examined the genetic architecture of energy expenditure (EE) in genome-wide linkage scans [16–18], admixture mapping [19], or candidate gene analyses [20–24]. A single genome-wide association study (GWAS) has examined EE and related obesity phenotypes in Hispanic children [25]. However, it is difficult to amalgamate these studies since the phenotypic measures in these studies were conducted under various conditions (e.g., laboratory or free-living), and used different methods to assess RMR, other metabolic biomarkers, and physical activity characteristics. Additionally, it is likely that the common SNPs interrogated using GWAS and early candidate gene studies did not capture the effects of rare variants which may play a role in RMR.

The goal of this study was to use an exome array genotyping platform to identify genetic variants which might influence an individual’s RMR, and determine their association with body adiposity and metabolic biomarkers. We hypothesized that known obesity-related variants would also be associated with RMR. To test this hypothesis, we evaluated the genetic associations of exonic variation with RMR measured using reference standard room calorimetry in a racially diverse and carefully phenotyped cohort of children and adults. Additionally, we examined the most significant results of the RMR genetic analysis with adiposity measures, lipids, and glucose homeostasis, in a candidate phenome-wide association study, as well as using genetically predicted gene expression (GPGE) to identify gene targets of single nucleotide polymorphisms (SNPs) with an effect on gene expression in a variety of body tissues. Overall, our long-term goal is to locate genetic variants, which determine inter-individual differences in RMR that potentially can be used to alter lifestyle and treatment on an individual level.

## Methods

### Participants recruitment and study design

Healthy youth (8 ***–*** 17 years old) and adults (18 ***–*** 65 years old), with a range of BMI values were recruited from the Metropolitan Nashville, Tennessee, USA general population. Study participants were recruited using flyers, e-mail distribution lists, and personal contacts to studies evaluating novel methods to measure physical activity in youth and adults [26]. Descriptive characteristics of participants are presented in Table 1. All participants were healthy as determined by a physician, nonsmokers, without limitations on exercise tolerance, and without chronic pulmonary conditions (e.g. asthma) or taking prescription medications known to interfere with RMR. All applicable institutional and governmental regulations concerning the ethical use of human volunteers were followed during this study, in accordance with the ethical principles of the Helsinki-II Declaration. All adult (≥18 years old) and youth (<18 years old) participants and the parents or legal guardians of youth signed an informed consent or assent document as appropriate, which was approved by the Vanderbilt University Institutional Review Board.

**Table 1:** Characteristics of study samples by race/ethnicity

Characteristic	Total		White		Black		Hispanic	
	N	Mean[SD] or %	N	Mean[SD] or %	N	Mean[SD] or %	N	Mean[SD] or %
Age (years)	262	28[16]	148	30[16]	106	26[15]	7	23[11]
Sex (% female)	262	40%	148	44%	106	37%	7	29%
Weight (kg)	262	76[23]	148	76[23]	106	76[21]	7	80[17]
Height (m)	262	1.64[0.12]	148	1.65[0.13]	106	1.64[0.10]	7	1.65[0.12]
BMI (kg/m^2^)	262	28[7]	148	27[7]	106	28[7]	7	29[2]
Fat Mass (kg)	250	27[15]	142	27[15]	100	27[15]	7	31[7]
Fat-Free Mass (kg)	250	49[13]	142	49[14]	100	49[11]	7	49[17]
Body Fat %	250	34[12]	142	34[12]	100	35[13]	7	41[10]
SBP (mmHg)	225	119[14]	122	120[16]	95	120[11]	7	116[14]
DBP (mmHg)	225	72[9]	122	72[10]	95	71[8]	7	71[6]
Glucose (mg/dL)	165	50[38]	94	52[37]	65	45[39]	5	75[32]
Insulin (μU/mL)	222	73[62]	130	66[48]	86	81[65]	5	137[197]
Cholesterol (mg/dL)	228	163[34]	124	166[36]	98	159[31]	6	164[37]
Triglycerides (mg/dL)	228	81[57]	124	93[66]	98	65[37]	6	110[32]
HDL (mg/dL)	228	54[17]	124	52[16]	98	56[18]	6	43[18]
LDL (mg/dL)	228	93[29]	124	96[31]	98	89[25]	6	99[21]
RMR (kcal/day)	258	1912[532]	144	1885[515]	106	1940[536]	7	1898[722]
VO_2_Max (ml/kg/min)	184	2286[849]	96	2505[969]	84	2039[601]	4	2029[741]

Phenotypic assessments were conducted at the Clinical Research Center (CRC) during two study visits. At the first visit, sociodemographic information including self-declared race/ethnicity, health history, systolic and diastolic blood pressure, and physical fitness (VO_2max_) were assessed. The second visit included measurement of body composition using dual-energy X-ray absorptiometry (DXA) and a ~24-h stay in a fast response, whole-room indirect calorimeter (volume = 19 m^3^) that assures high-precision EE measurements in a controlled environment under semi-naturalistic conditions (i.e. not wearing a breathing mask). During the stay, participants followed a structured protocol consisting of self-paced 10-min ambulatory exercises and sedentary and activity tasks throughout morning and afternoon sessions. Beyond these structured activities, participants were encouraged to resume their normal daily routine as much as possible without specific suggestions. The meals (breakfast, lunch, dinner, afternoon and evening snacks) were given at set times and portions were individualized for body weight and activity level energy content and macro- and micronutrient amounts. Participants were instructed to go to bed at 10:00 pm and were woken up at 6:00 am for the measurement of RMR. After RMR measurement, a fasting blood sample was collected for measuring physiological markers and DNA extraction. After eating breakfast, participants were discharged from the CRC.

### Data collection protocol

#### Phenotyping

Stature (height) was measured within 0.5 cm using a calibrated wall-mounted stadiometer (Perspective Enterprises, Portage, MI). Body weight was measured within 0.1 kg using a calibrated beam platform scale (Detecto-Medic, Detecto Scales, Inc, Northbrook, IL) with participants wearing light clothing and no shoes. BMI was calculated from weight and height (kg/m^2^). Total body fat free mass and fat mass were measured using DXA (GE Medical Systems, Madison WI, enCORE 2007 software version 11.40.004). Systolic and diastolic blood pressure (SBP and DBP, respectively) were measured in triplicate after 10 min of resting in a supine position using an automatic inflating blood pressure cuff (DINAMAP, GE Healthcare). RMR was measured in the room calorimeter in the morning following an overnight sleep and a 10-h fast, and was defined as the average EE during a 30-min period while the subject laid in the supine position with minimal movement. RMR was calculated minute-by-minute from measured rates of oxygen (O_2_) consumption and carbon dioxide (CO_2_) production using Weir’s equation [27]. Peak oxygen uptake (VO_2max_) was measured using a modified Bruce treadmill exercise test protocol [28]. Breath-by-breath O_2_ consumption and CO_2_ production were measured using a MedGraphics Ultima Series system, and processed and analyzed with the BreezeSuite software Version 6.4.023 (St. Paul, MN).

#### Blood collection and measurements

Blood samples were collected following ~10 h of sleep and fast in the room calorimeter. Plasma was separated by centrifugation and stored at -80 °C. Glucose was measured using the Vitros Chemistry analyzer and insulin was measured using radioimmuno assay. Plasma triglycerides, total cholesterol, low-density lipoprotein (LDL), and high-density lipoprotein (HDL) concentrations were measured using enzymatic kits from Cliniqa Corp. (San Marcos, CA).

### DNA extraction and genotyping

All DNA samples were isolated from whole blood using the Autopure LS system (QIAGEN Inc., Valencia, CA). Genomic DNA was quantitated via an ND-8000 spectrophotometer and DNA quality was evaluated via gel electrophoresis. We genotyped DNA from the 272 participants using the custom Affymetrix Axiom Exome Genotyping Array (Affymetrix Inc., Santa Clara, CA). The genomic DNA samples were processed according to standard Affymetrix procedures for processing of the assay. The data were processed for genotype calling using the Affymetrix Power Tools software (APT, Affymetrix Inc., Santa Clara, CA).

### Genotyping quality control

All monomorphic single nucleotide polymorphisms (SNPs) (*N* = 163,778) were removed. Variants were retained for analysis if they had a minor allele frequency (MAF) of at least 0.01 and did not deviate from Hardy-Weinberg Equilibrium (*p* > 1 × 10^−6^). This resulted in 66,088 variants for further analysis. Quality control also removed three individuals for low genotyping efficiency (genotyping call rate <95%), and seven individuals were removed due to gender errors (using only those variants with a MAF >0.2), for a final total of 262 individuals. In order to quantify ancestry among samples, EIGENSTRAT [29] was used to estimate continuous axes of ancestry. The top five principal components (PCs) were used as covariates in regression models to test for genotype associations.

### Statistical analysis

Demographic data were presented as means and standard deviations for continuous variables, and frequencies and proportions for categorical data, and analyzed using linear regression (STATA 14.0 statistical software, College Station, TX). Single variant linear regression analysis was performed on the entire sample using the RVTest analysis program and incorporating information from a kinship matrix to account for relatedness between subects [30]. The results were processed using RAREMETAL [31] for single variant and SKAT gene-based tests [32] and construction of summary figures. Effect sizes are reported as regression coefficients and standard errors. RMRs were inversely transformed. Analyses included age, sex, five principal components as covariates to address the ethnic heterogeneity, and BMI to account for the impact of body size on RMR. Statistical significance was determined using a Bonferroni correction (*p*-value threshold = 7.57 × 10^−7^ for single variant analysis).

For the candidate PheWAS, top SNPs from the association analysis with RMR were selected for evaluation with other phenotypes available in the sample. Phenotypes were transformed to approximate normality, if necessary. BMI was excluded as a covariate for measures directly related to body composition (i.e. fat mass, fat-free mass, body fat percentage). Statistical analysis was performed as above, using RVTest and RAREMETAL.

To evaluate the effects of known obesity genes on RMR, we examined all variants within significant genes reported in the Locke et al. [33] (BMI), Shungin et al [34] (waist-to-hip ratio adjusted for BMI), and Lu et al [35] (body fat percentage) GWAS papers for evidence of association, assuming an a priori hypothesis of association between obesity-risk genes and RMR.

To evaluate the genetic association results in the context of gene expression further, we employed a novel MetaXcan method [36], which conducts a test of association between phenotypes and gene expression levels predicted by genetic variants in a library of tissues from the GTEx project [37]. MetaXcan is a meta-analysis approach that conducts the PrediXcan [38] test using genotype association summary statistics, rather than conducting the tests in individual level data.

## Results

**Fig. 1:**
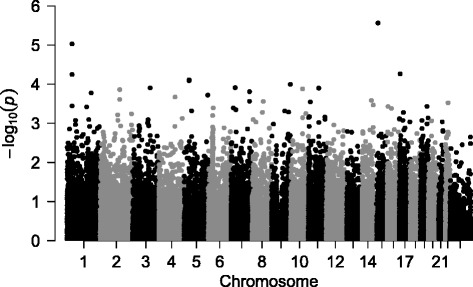
Manhattan plot of single variant associations with resting metabolic rate

The sample consisted of 118 children (ages 10–16) and 159 adults (ages 18–67). A majority (*n* = 158; 57%) were European Americans, 40% were African Americans (n = 106), and 3% were Hispanics (n = 7; Table 1). Daily RMR was higher among children than adults (1994.0 vs 1782.4; *p*-value = 0.0005), correlated with fat-free mass (*r* = 0.62 and 0.56, respectively) and correlated with body weight more strongly in children than adults (*r* = 0.70 and 0.32, respectively; Tables S1 and S2 (in Additional file 1)). BMI was not strongly correlated with RMR in adults (*r* = 0.20), but was more so in children (*r* = 0.57), therefore genetic association with RMR was evaluated both with and without BMI as a covariate. Although single variant association testing for RMR adjusted for age, sex, BMI and principal components of ancestry (lambda = 1.041; Figure S1 (in Additional file 1)) did not reveal any signals reaching stringent statistical significance thresholds (Table 2), the top signal (*p*-value = 2.73 × 10^−6^) we found is located on chromosome 15 (Fig. 1). This variant, rs74010762, leads to a synonymous change within the *TRPM1* gene (transient receptor potential cation channel, subfamily M, member 1). Additional findings of potential interest include two nonsynonymous variants in *SH3D21* (SH3 [SRC Homology 3] domain containing 21). Both SNPs (rs2358728 and rs2358729) are uncommon, each with an MAF of 0.05. BMI unadjusted results are presented in Additional file 1: Figures S2–S5 .

**Table 2:** Top results from single variant association analysis of inverse RMR

SNP	Chr.	Position	Alleles	Annotation	MAF	*P*-value	Beta	Beta SE
rs74010762	15	31362279	A/G	Synonymous:*TRPM1*	0.02	2.7E-06	−0.00015	3.2E-05
rs2358728	1	36785855	C/T	Nonsynonymous:*SH3D21*	0.05	9.4E-06	−0.00011	2.5E-05
rs200903851	17	5353492	-/A	Intron:*DHX33*	0.01	5.5E-05	0.00019	4.8E-05
rs2358729	1	36785853	C/A	Nonsynonymous:*SH3D21*	0.05	5.7E-05	−0.00011	2.7E-05
rs115795863	5	36227609	T/C	Nonsynonymous:*C5orf33*	0.03	7.7E-05	0.00013	3.2E-05
rs35433829	5	36265559	G/T	Nonsynonymous:*RANBP3L*	0.03	8.1E-05	0.00013	3.2E-05
rs28545754	9	140243844	C/T	Nonsynonymous:*EXD3*	0.45	0.00010	3.7E-05	9.4E-06
rs8192498	7	30701812	C/T	Nonsynonymous:*CRHR2*	0.02	0.00012	0.00014	3.7E-05
rs41272321	3	132338346	T/G	Nonsynonymous:*ACAD11*	0.08	0.00013	−6.8E-05	1.8E-05
rs2729772	11	76979550	T/C	Nonsynonymous:*GDPD4*	0.10	0.00013	5.5E-05	1.4E-05
rs41284084	10	90492227	T/G	Nonsynonymous: *LIPK*	0.06	0.00013	8.7E-05	2.3E-05
rs1484930	2	146259628	C/A	Intergenic	0.32	0.00014	−4.9E-05	1.3E-05
rs2251220	7	138601826	G/A	Nonsynonymous:*KIAA1549*	0.48	0.00016	4.2E-05	1.1E-05
rs10752838	1	181116023	C/T	Intergenic	0.42	0.00017	−3.9E-05	1.1E-05
rs924752	4	120853499	A/G	Intergenic	0.46	0.00021	−3.9E-05	1.0E-05
rs6430083	2	146247003	T/C	Intergenic	0.32	0.00025	−4.7E-05	1.3E-05
rs2401751	14	88946622	G/A	Nonsynonymous:*PTPN21*	0.36	0.00026	−4.5E-05	1.2E-05
rs2774960	7	138602417	G/A	Nonsynonymous:*KIAA1549*	0.47	0.00027	4.0E-05	1.1E-05
rs73277676	8	83110968	T/G	Intergenic	0.16	0.00028	−5.7E-05	1.6E-05
rs9630182	11	13620172	C/T	Intergenic	0.45	0.00028	−3.8E-05	1.0E-05
rs80575	22	36539754	T/G	Intron:*APOL3*	0.48	0.00030	−3.9E-05	1.1E-05

In our analysis of primary traits of interest among the other available metabolic and anthropometric phenotypes in a candidate PheWAS (phenome-wide association study) we found a few nominally significant associations (Table 3). The most significant association was between rs73277676 (RMR *p*-value = 2.8 × 10^−4^) and systolic blood pressure (*p*-value = 0.003). Cross-phenotype comparison of the two *SH3D21* variants revealed nominal associations with glucose level (*p*-values = 0.032 and 0.039). The overall top variant from the RMR single variant analysis, rs74010762, was not significantly associated with any of the phenotypes evaluated, nor did any variant reach statistical significance accounting for the number of SNPs and phenotypes tested (*p*-value < 1.90 × 10^−4^), albeit a conservative threshold given the correlation between phenotypes. Fat free mass, fat mass, and bone mineral density were associated with a small number of variants approaching suggestive significance thresholds. Glucose level was associated with most of the candidate SNPs, with 5 SNPs having a *p*-value of ≤0.05.

**Table 3:** Candidate PheWAS p-values for RMR-associated variants with related traits

SNP	Body Fat %	Fat Mass	Fat-Free Mass	BMI	Glucose	Insulin	Total Cholesterol	LDL	HDL	Trig.	VO_2_Max	SBP
rs74010762	0.96	0.36	0.061	0.36	0.44	0.13	0.44	*0.050*	0.13	0.51	0.24	0.53
rs2358728	0.30	0.72	0.088	0.95	*0.032*	0.14	0.66	0.53	0.43	0.14	0.38	0.90
rs200903851	0.66	0.60	0.26	0.52	0.24	0.90	0.58	0.085	0.22	0.74	*0.039*	0.19
rs2358729	0.88	0.75	0.16	0.69	*0.039*	0.24	0.98	0.99	0.59	0.19	0.65	0.90
rs115795863	0.30	0.29	0.77	0.81	*0.050*	0.86	0.95	0.70	0.60	0.52	0.55	0.20
rs35433829	0.29	0.28	0.78	0.81	*0.050*	0.86	0.93	0.67	0.59	0.51	0.57	0.20
rs28545754	0.14	0.33	0.14	0.91	0.72	0.90	0.46	0.43	0.76	0.34	0.22	0.98
rs8192498	0.29	0.49	0.68	0.59	0.96	0.077	0.43	0.99	0.83	0.090	0.43	0.42
rs41272321	0.69	0.38	0.93	0.53	0.87	0.20	*0.014*	*0.028*	0.24	0.59	0.99	0.94
rs2729772	0.68	0.50	0.38	0.41	*0.014*	0.064	0.35	0.81	0.62	0.23	0.43	*0.050*
rs41284084	0.12	0.21	0.89	0.38	0.95	0.32	0.11	0.32	0.48	0.92	0.23	0.61
rs1484930	0.55	0.79	0.21	0.87	0.59	0.066	0.62	0.81	0.70	0.45	0.16	0.091
rs2251220	0.68	0.95	0.31	0.84	0.81	0.37	0.57	0.22	0.052	0.16	0.21	0.85
rs10752838	0.66	0.93	0.33	0.41	0.79	*0.027*	0.25	0.58	0.56	0.59	0.061	0.19
rs17624798	0.90	0.81	0.38	0.50	0.91	0.33	0.48	0.81	0.26	0.46	0.26	0.95
rs924752	0.77	0.62	0.25	0.37	0.68	0.17	0.89	0.96	0.27	0.43	0.12	0.87
rs6430083	0.46	0.71	0.23	0.74	0.85	0.066	0.62	0.82	0.76	0.31	0.14	0.058
rs2401751	0.45	0.56	0.25	1.00	0.28	0.43	0.48	0.48	0.37	0.11	0.47	0.71
rs2774960	0.86	0.74	0.15	0.89	0.90	0.77	0.64	0.30	0.052	0.12	0.21	0.94
rs73277676	0.47	0.46	0.88	0.53	0.089	0.22	0.41	0.55	0.61	0.56	*0.046*	*0.0038*
rs9630182	0.41	0.38	0.85	0.98	0.13	0.21	0.17	0.11	0.73	0.47	0.62	0.22
rs80575	0.75	0.082	*0.046*	0.10	0.79	0.47	0.88	0.71	0.77	0.75	0.082	0.20

*LDL* Low density lipoprotein, *HDL* high density lipoprotein, *Trig*. Triglycerides, *SBP* systolic blood pressure

**Fig. 2:**
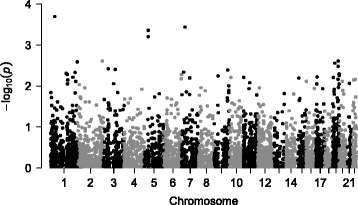
Gene-based analysis for resting metabolic rate

Results of gene-based association tests using SKAT for all variants with a MAF less than 5% are presented in Table 4 and Fig. 2. The most strongly associated gene with RMR was *SH3D21* (*p*-value = 2 × 10^−4^), represented by the two variants mentioned above. Other genes of interest included *CRHR2* (corticotropin releasing hormone receptor 2) and *RANBP3L* (RAN binding protein 3-like). Both of these genes were represented by one variant among the top hits from the single variant analysis and each had two total SNPs for the SKAT analysis.

**Table 4:** Gene-based tests of resting energy expenditure

Gene	Chr.	Variants	Average Allele Frequency	Minimum Allele Frequency	Maximum Allele Frequency	*P*-Value
*SH3D21*	1	2	0.048	0.047	0.049	2.0 × 10^−4^
*CRHR2*	7	2	0.031	0.019	0.043	3.6 × 10^−4^
*RANBP3L*	5	2	0.031	0.031	0.031	4.4 × 10^−4^
*C5orf33*	5	2	0.020	0.0097	0.031	6.2 × 10^−4^

To best utilize the exome chip, which emphasizes coverage of coding variants, and to account for differences from reported index variants, we evaluated all variants within known obesity-related genes from the GWAS catalog (270 variants from 79 genes) in the context of RMR. Evaluation of these variants did not yield substantially more nominally significant associations than expected by chance (0.05 × 270 = 13.5; Table S2 (in Additional file 1)). The top result rs35433754 (*p*-value = 0.0017) was in the gene *TNKS*, previously implicated in extreme early-onset obesity and adult waist circumference [39]. Despite the genes being chosen for known associations with obesity [33], adjusting for BMI did not affect the estimates of effect or significance of the test in most cases (11/17).

Evaluation of genetically predicted gene expression (GPGE) levels in GTEx tissue references identified *UHMK1* (U2AF homology motif (UHM) kinase 1) GPGE in tibial nerve tissue as nominally associated with RMR by MetaXcan (*p*-value = 5.96 × 10^−5^; Table 5). This gene is not near any of the top association results presented in Table 2. Also notable from the GPGE results are the number of tissues (*N* = 16) in which *ACP6* (acid phosphatase 6, lysophosphatidic) predicted expression was associated with *p*-value <0.0001.

**Table 5:** Genetically predicted gene expression results from MetaXcan with RMR genetic association data

Gene	Z-score	*P*-value	r^2^	Tissue
*UHMK1*	4.01	5.96E-05	0.056	Nerve-Tibial
*ZNF506*	−3.84	0.00012	0.021	Skin-SunExposed
*B3GNT6*	−3.80	0.00014	0.18	Brain_Hippocampus
*RTN2*	3.74	0.00018	0.028	Brain_Hypothalamus
*ZNF682*	−3.72	0.00019	0.025	Colon_Sigmoid
*RAB5C*	−3.67	0.00024	0.023	Heart_AtrialAppendage
*UBN2*	−3.66	0.00026	0.048	Artery_Aorta
*GALC*	3.65	0.00026	0.051	Brain_Cortex
*SPATA7*	−3.65	0.00026	0.012	Pituitary
*ZC3HAV1L*	3.64	0.00027	0.062	Stomach
*FAR1*	−3.63	0.00028	0.015	Brain_Anteriorcingulatecortex
*PVALB*	−3.62	0.00030	0.026	Artery_Aorta
*PVALB*	3.62	0.00030	0.079	Brain_CerebellarHemisphere
*FMO5*	3.55	0.00038	0.13	Adrenal
*ACP6*	3.55	0.00038	0.00037	Brain_CerebellarHemisphere
*ACP6*	3.55	0.00038	0.47	Brain_Cortex
*ACP6*	3.55	0.00038	0.39	Brain_FrontalCortex
*ACP6*	3.55	0.00038	0.23	Brain_Hippocampus
*ACP6*	3.55	0.00038	0.43	Brain_Putamen_basalganglia
*ACP6*	3.55	0.00038	0.36	Cells_fibroblasts
*ACP6*	3.55	0.00038	0.26	Colon_Sigmoid
*ACP6*	3.55	0.00038	0.40	Colon_tranverse
*ACP6*	3.55	0.00038	0.34	Esophagus_GastroesophagealJunction
*ACP6*	3.55	0.00038	0.37	Esophagus_Muscularis
*ACP6*	3.55	0.00038	0.57	Pituitary
*ACP6*	3.55	0.00038	0.12	SmallIntestine
*ACP6*	3.55	0.00038	0.39	Stomach
*ACP6*	3.55	0.00038	0.48	Testis
*ACP6*	3.55	0.00038	0.52	Thyroid
*BCL9*	3.55	0.00038	0.0050	WholeBlood
*ACP6*	3.53	0.00041	0.17	Brain_Cerebellum
*RP11-345 J4.3*	−3.52	0.00043	0.052	Artery_Tibial
*HABP2*	−3.49	0.00048	0.012	Esophagus_GastroesophagealJunction
*KRT38*	3.49	0.00048	0.28	WholeBlood
*GPX8*	−3.49	0.00048	0.00093	Pituitary
*PPAP2A*	−3.49	0.00048	0.0018	Spleen
*ZNF169*	−3.49	0.00048	0.031	Brain_Cerebellum
*BARX1*	3.49	0.00048	0.021	Brain_Cortex
*PTPDC1*	−3.49	0.00048	0.040	Esophagus_GastroesophagealJunction

## Discussion

This is the first study assessing association of exome chip gene coding variation with RMR measured using a reference standard method in a in a multi-ethnic sample of children and adults. We evaluated 66,088 genetic variants in 262 individuals through single-variant association and gene-based tests of genetic exposures and GPGE, and also considered the effect of known obesity loci. We did not identify a variant or a gene associated with RMR and meeting stringent Bonferroni statistical significance thresholds. However, we identified some suggestive associations, which may have biological plausibility for a role in energy homeostasis and merit further studies.

The *TRPM1* (transient receptor potential cation channel, subfamily M, member 1) gene showed the most significant association with RMR. This gene has been previously implicated in Mendelian forms of night blindness [40–42], and functionally localized to retinal cells [43, 44]. Expression of this gene in GTEx suggests other tissues with high expression are skin and testis. The functional role of the *SH3D21* gene significantly associated with RMR is not well known aside from containing three SH3 domains suggesting a likely role in protein binding. However, among tissues included in GTEx, *SH3D21* is most robustly expressed in thyroid.

Other genes of interest included *CRHR2* (corticotropin releasing hormone receptor 2) and *RANBP3L* (RAN binding protein 3-like). *CRHR2* has been associated with endometriosis [45], localization of glucose transporters in the placenta [46], colorectal cancer [47], stress response in the cortisol pathway [48, 49], and leptin responsiveness [50]. Variants in this gene have also been associated with preterm birth [51]. This gene is expressed predominantly in the pituitary gland from GTEx. RANBP3L has been observed to act as a nuclear exporter for Smad1/5/8, however, little else has been reported to date about this protein [52]. Expression of this gene in GTEx is observed more predominantly in brain regions, including caudate and nucleus accumbens. Genetic analyses have identified suggestive associations near *RANBP3L* with height [53], hypertension [54], and serum tamsulosin hydrochloride concentration [55]. Although the SNP associated with both RMR and systolic blood pressure is located in an intergenic region, there were also several nominal signals among RMR hits that were associated with glucose levels. These five variants include two in *SH3D21*, one in *RANBP3L*, one in *GDPD4* (glycerophosphodiester phosphodiesterase domain containing 4) and one in *NADK2*, which is a mitochondrial NAD kinase. These PheWAS results would suggest that RMR is a complex phenotype, and the genetic underpinnings of this trait share some modest effects with other metabolic and anthropometric traits.

Evaluation of obesity-related genes for association with RMR suggested a role for variants in *PLA2G6, NEGR1,* and *NRXN3* (Table S2 (in Additional file 1))*.* Each of these genes had multiple variants nominally associated with RMR. All of these genes are well established with regard to associations with BMI and obesity [56–58]. *PLA2G6* is expressed most in the thyroid, while *NRXN3* and *NEGR1* have expression patterns predominantly in brain (frontal cortex, cerebellar hemisphere (both genes), cerebellum (*NRXN3*), and cortex (*NEGR1*)).

GPGE results implicated five established genes related to obesity, body composition or glucose metabolism (Table 5). Two of the results, *BARX1* (BARX homeobox 1) and *PTPDC1* (protein tyrosine phosphatase domain containing 1), represent a single associated locus for waist-to-hip ratio from the most recent GIANT consortium meta-analysis [34], while *ZNF169* (zinc finger protein 169) was implicated at a suggestive p-value with BMI in East Asians [59]. Further, variants near *FAR1* (fatty acyl-CoA reductase 1) have been previously associated with bone mineral density in Hispanic children [25], and *BCL9* has recently been identified with type 2 diabetes in aboriginal Australians [60]. *FAR1* has also been shown to be expressed differently between visceral and subcutaneous adipose tissue in colorectal cancer patients [61]. Of these GPGE association results, *BARX1*, *ZNF169* and *FAR1* were all significant in brain regions, which is consistent with a popular hypothesis that the central nervous system regulation of energy balance plays a major role in the development of obesity [62–65].

The most significant GPGE association of RMR was with the *UHMK1* (U2AF homology motif (UHM) kinase 1) gene. Genetic variants in *UHMK1* have been associated with schizophrenia [66–68], and gene expression evaluations have been performed in mouse brain [69] with particular interest in pharmacological treatment effects [70]. Interestingly, both *UHMK1* and *ACP6* (acid phosphatase 6, lysophosphatidic) have been implicated in cerebral vision impairment from exome sequencing [71]. *ACP6* GPGE was significant in several brain regions (cerebellar hemisphere, cerebellum, cortex, frontal cortex, hippocampus, and Putamen basal ganglia), the pituitary and thyroid glands, several tissues in the gastrointestinal system (stomach, small intestine, sigmoid and transverse sections of the colon, as well as muscularis and gastroesophageal junction of the esophagus). Among the top results, brain regions were somewhat over-represented (13/39 results = 0.33) compared to the number of tissues evaluated (10/40 tissues = 0.25). Three of the results were in the pituitary gland (*SPATA7, ACP6*, and *GPX8)* which has implications with growth hormone levels, implying a relationship with energy balance in children.

Few other studies have analyzed genetic variants underlying RMR and related phenotypes measured by whole-room calorimetry, particularly in children, on a genome-wide scale. Due to the novel design, we considered a separate evaluations of children, however, the sample size (and therefore power) is reduced dramatically in this case. We have included the results for children alone in the supplementary material (Figures S6–S9; Table S3 (in Additional file 1)), but with a sample size of only 112, we do not feel confident in these results as a stand-alone analysis. The Viva La Familia study, did evaluate genome-wide genetic associations with obesity-related traits [25]; however, those did not replicate here (data not shown). Likely reasons for the lack of replication is the vastly different genotyping arrays with little overlap, but this is also potentially contributed to by the difference in ethnic origin and the small sample size of this study. Additionally, the phenotypes assessed were not entirely consistent between the two studies, with the GWAS in Viva La Familia evaluating total and sleep EE rather than the RMR phenotype considered here, though both studies made use of whole-room indirect calorimetry. Basal energy expenditure was assessed in their study [16], utilizing the same method as our RMR measure; however, this phenotype characteristic was not included in their GWAS publication.

Previous linkage scans for RMR have implicated regions 16q22.312, 3q26.114, and 11q23-q2413 which do not overlap with the regions identified in this study. The region on chromosome 16 harbors many variants implicated in a wide variety of disorders, including multiple sclerosis [72], breast cancer [73], hypospadias [74], and atrial fibrillation [75–77], among others, though none are directly related to energy balance or obesity. Another region detected in the Quebec Family Study, 22q12.314, does harbor the gene *APOL3*. One SNP in this gene was nominally associated with RMR in the present study, although not reaching robust statistical significance thresholds (*p*-value = 3.0 × 10^−4^). Other genes in this region have also been implicated in differential adipose deposition (*LARGE* [78] and *HMGXB4* [34]) and fat mass (near *ODF3B*) [25], and electronic medical record-defined BMI in children (*APOL5*) [79], suggesting that our study has detected a biological relationship with energy balance or storage in this region, consistent with these previous studies.

## Conclusions

In summary, this is the first large-scale genetic association study of RMR in African- and European-American children and adults incorporating GPGE data. The results suggest that several obesity and body composition-related loci are also associated with RMR, and highlight the role of PheWAS in evaluating the phenotypic spectrum associated with selected genetic variants. Our findings of previously unknown signals may suggest that RMR is incompletely explained by anthropometrics, glucose metabolism and energy balance genetic variants. Although large-scale association studies combining accurate RMR measurements with comprehensive phenotyping are challenging, their results might provide information for research focused on precision medicine in individuals. In conclusion, our results suggest that RMR may be partially independent of anthropometric phenotypes, and that genetic evaluation of this trait provides evidence supporting a role for RMR in obesity pathophysiology.

## Additional file

Additional file 1: Brief description: Supplemental figures and tables including quantile-quantile plots for main and secondary analyses, manhattan plots of secondary analyses (without BMI adjustment, children only), and correlation tables. (PDF 632 kb)

## Abbreviations

BMI: Body mass index
CRC: Clinical Research Center
DBP: Diastolic blood pressure
DXA: Dual-energy X-ray absorptiometry
EE: Energy expenditure
GPGE: Genetically predicted gene expression
GWAS: Genome-wide association study
HDL: High density lipoprotein
LDL: Low density lipoprotein
MAF: Minor allele frequency
PC: Principal component
PheWAS: Phenome-wide association study
RMR: Resting metabolic rate
SBP: Systolic blood pressure
SNP: Single nucleotide polymorphism
VO_2_Max: Peak oxygen uptake
