# Supplementary material for: Association of gene coding variation and resting metabolic rate in a multi-ethnic sample of children and adults
Source: BMC Obes. 2017 Apr 5;4:12. doi: 10.1186/s40608-017-0145-5 (PMC5381071; doi:10.1186/s40608-017-0145-5)
Supplement: Additional file 1: — Brief description: Supplemental figures and tables including quantile-quantile plots for main and secondary analyses, manhattan plots of secondary analyses (without BMI adjustment, children only), and correlation tables. (PDF 632 kb) [file 40608_2017_145_MOESM1_ESM.pdf]

Supplemental Data

Supplementary Figure 1. QQ plot for RMR adjusted for BMI

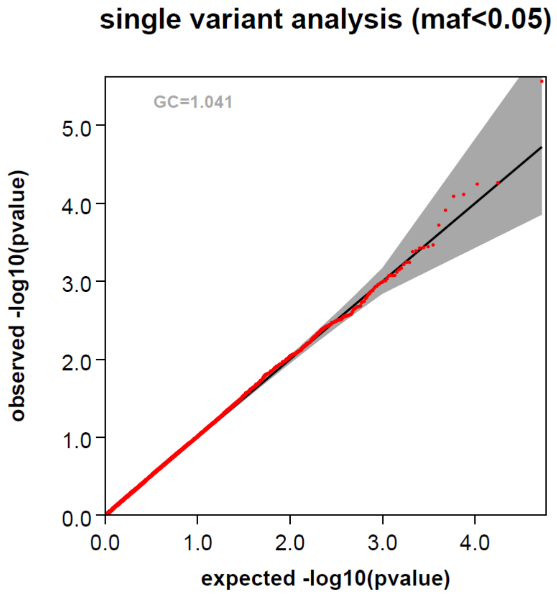

Supplementary Figure 2. QQ plot for RMR without adjustment for BMI

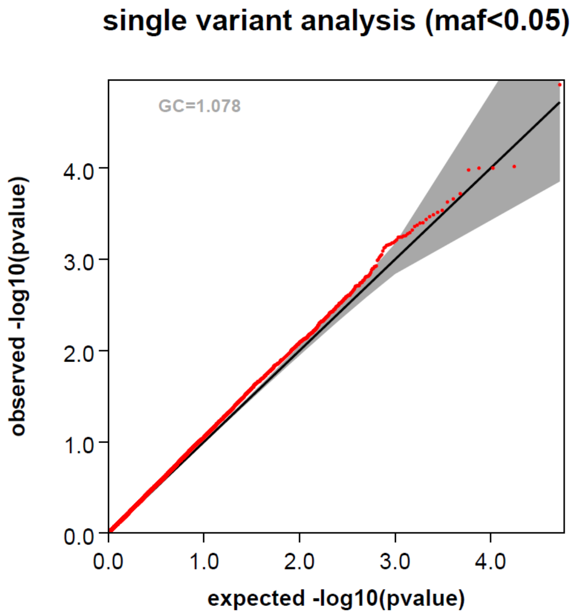

Supplementary Figure 3. Manhattan plot of RMR Association not adjusted for BMI

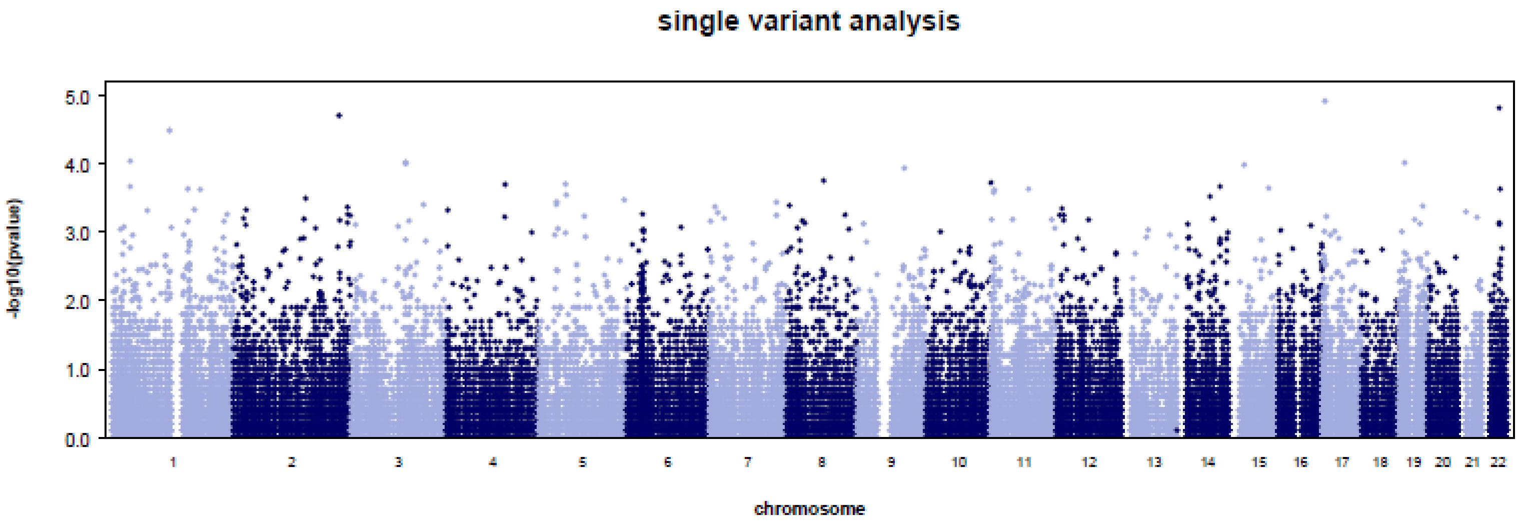

Supplementary Figure 4. QQ plot for gene-based association of RMR without adjustment for BMI

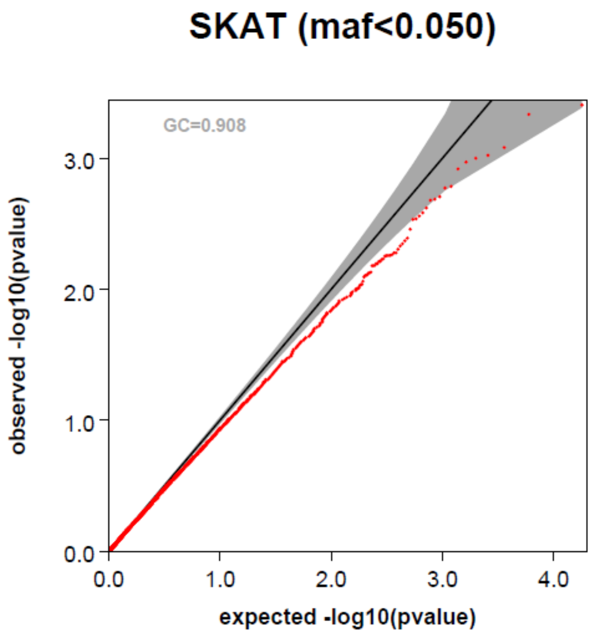

Supplementary Figure 5. Manhattan plot for gene-based association of RMR without adjustment for BMI

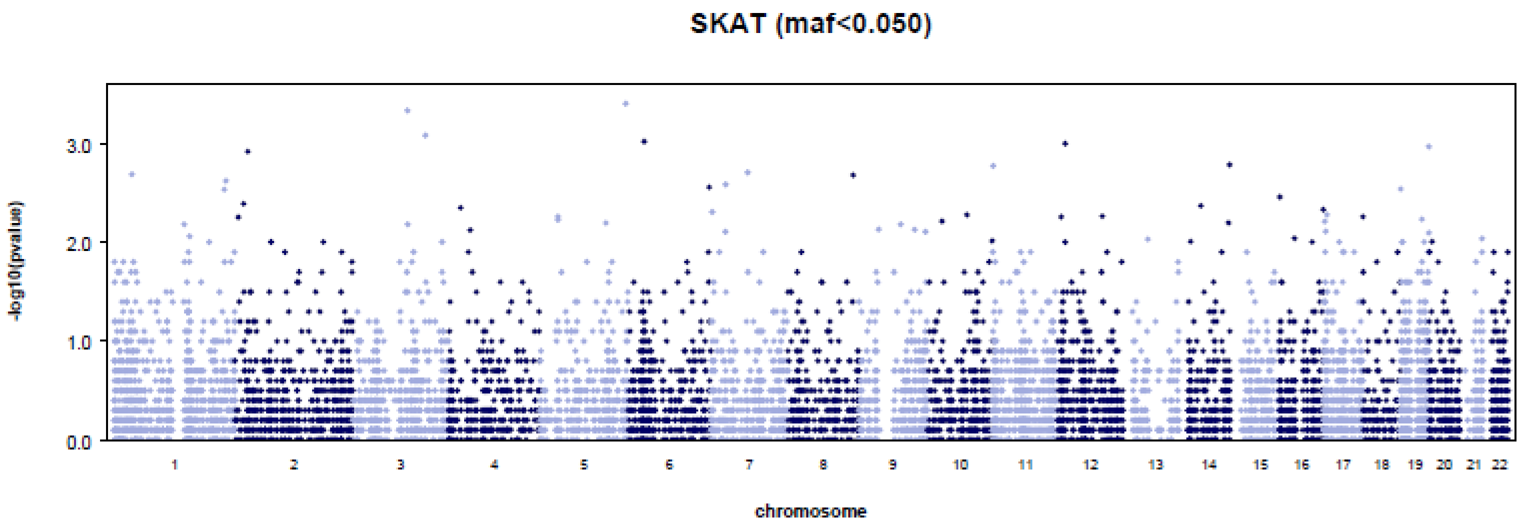

Supplementary Figure 6. RMR Single Variant analysis in Children (no BMI adjustment)

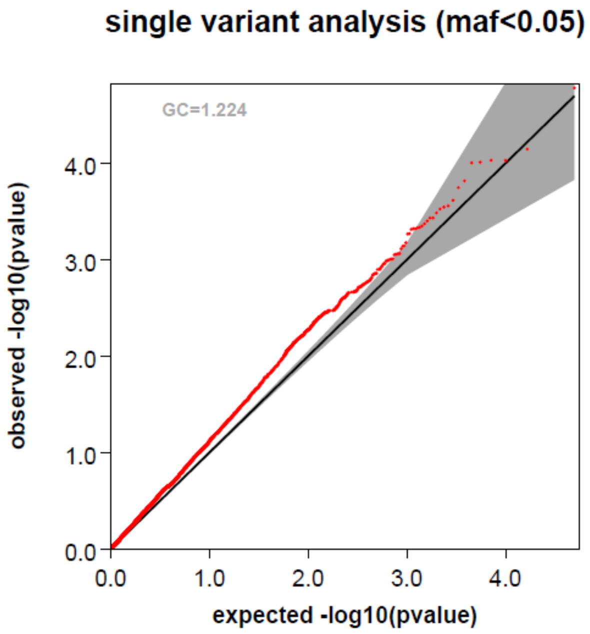

Supplementary Figure 7. RMR Single Variant Analysis in Children only (Not BMI adjusted)

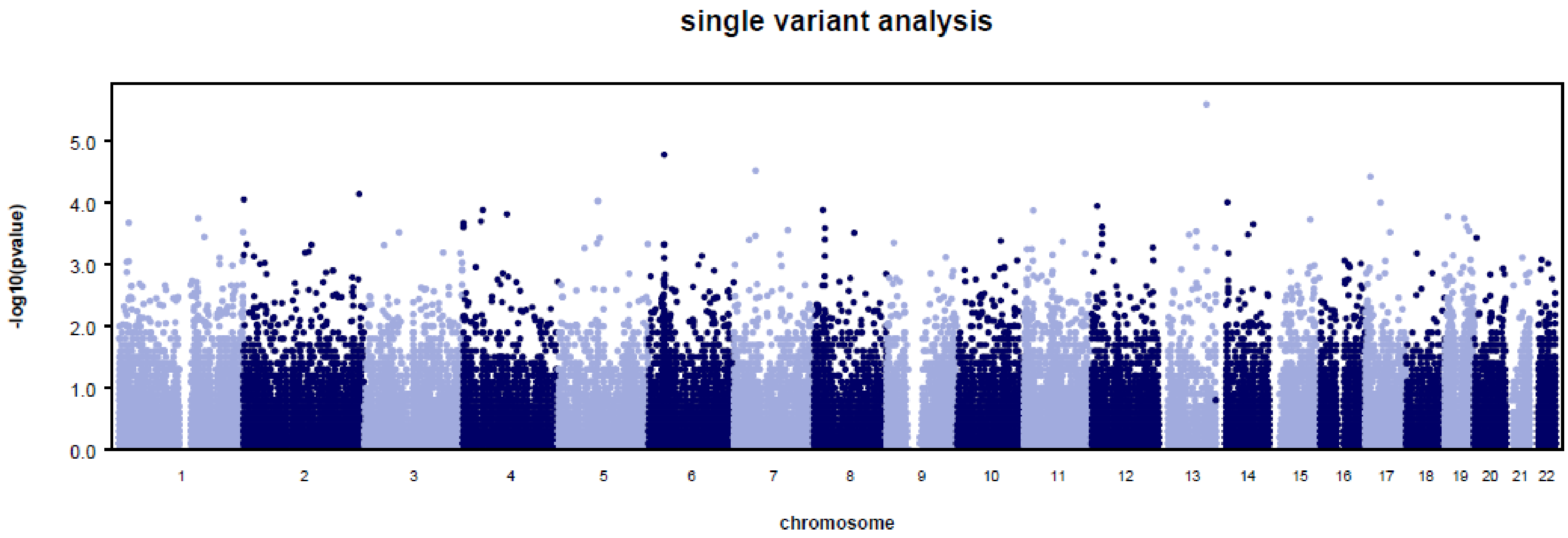

Supplementary Figure 8. SKAT analysis of RMR (not BMI adjusted) in Children only – qq plot

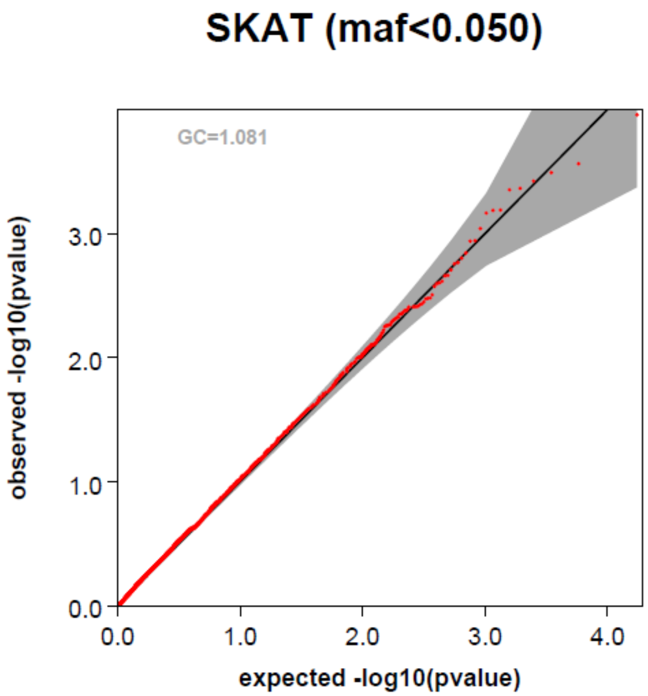

Supplementary Figure 9. SKAT analysis of RMR (not BMI adjusted) in Children only

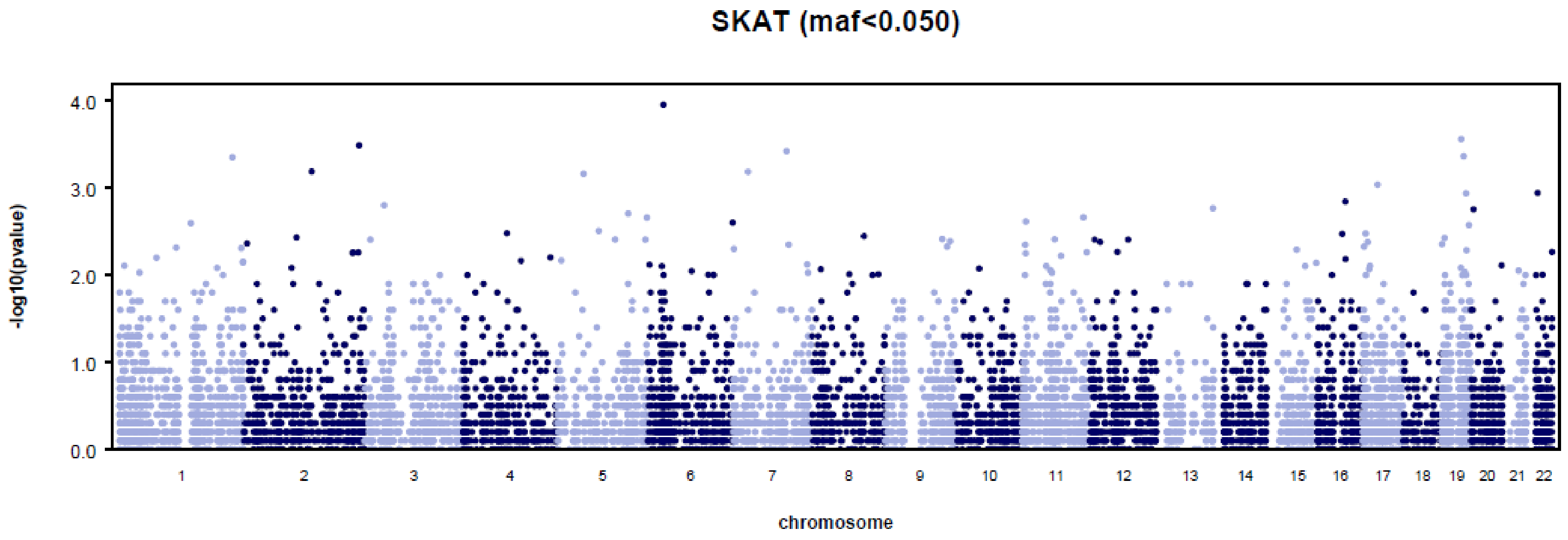

Supplementary Table 1. Correlation of measured phenotypes in adults

	Weight	Height	BMI	FatMass	BodyFat	FatFre~s	BMD	SBP	Insulin	Choles~l	Triglyc	HDL	LDL	REE	VO2Max	Glucose
Weight	1															
Height	0.406	1														
BMI	**0.760**	-0.269	1													
FatMass	**0.761**	0.012	**0.760**	1												
BodyFat	0.410	-0.278	**0.591**	**0.863**	1											
FatFreeMass	**0.690**	**0.651**	0.288	0.055	-0.332	1										
BMD	**0.645**	0.324	0.459	0.344	0.052	**0.610**	1									
SBP	0.344	0.229	0.186	0.215	0.063	0.318	0.214	1								
Insulin	0.248	-0.044	0.273	0.284	0.208	0.036	0.130	-0.046	1							
Cholesterol	0.187	-0.106	0.231	0.245	0.273	0.010	-0.009	0.160	-0.252	1						
Triglyc	0.370	0.177	0.227	0.306	0.204	0.236	0.203	0.199	-0.031	0.448	1					
HDL	-0.449	-0.231	-0.268	-0.344	-0.175	-0.268	-0.152	-0.129	-0.268	0.155	-0.399	1				
LDL	0.343	-0.048	0.338	0.368	0.337	0.071	0.000	0.183	-0.105	**0.849**	0.366	-0.299	1			
RMR	0.319	0.258	0.198	-0.069	-0.282	**0.559**	0.381	0.289	-0.133	0.025	0.068	-0.030	0.012	1		
VO2Max	0.262	**0.541**	-0.071	-0.257	**-0.597**	**0.626**	0.258	0.152	-0.223	0.000	0.188	-0.136	0.029	0.368	1	
Glucose	**0.598**	0.260	**0.620**	**0.667**	**0.623**	0.332	0.421	0.332	-0.120	**0.665**	0.366	-0.180	**0.594**	0.267	0.200	1

Correlations greater than |0.5| are in bold, with correlations greater than |0.7| also shaded in gray.

Supplementary Table 2. Correlation of measured phenotypes in children

	Weight	Height	BMI	FatMass	BodyFat	FatFre~s	BMD	SBP	Insulin	Choles~l	Triglyc	HDL	LDL	REE	VO2Max	Glucose
Weight	1															
Height	**0.633**	1														
BMI	**0.901**	0.249	1													
FatMass	**0.840**	0.202	**0.938**	1												
BodyFat	**0.548**	-0.094	**0.750**	**0.887**	1											
FatFreeMass	**0.802**	**0.860**	**0.525**	0.350	-0.031	1										
BMD	**0.706**	**0.651**	**0.533**	0.399	0.137	**0.778**	1									
SBP	**0.572**	0.418	0.485	0.406	0.189	**0.543**	0.408	1								
Insulin	-0.097	-0.165	-0.025	-0.049	-0.016	-0.105	-0.068	0.024	1							
Cholesterol	-0.126	-0.093	-0.122	-0.093	-0.062	-0.171	-0.129	0.006	-0.107	1						
Triglyc	0.141	-0.055	0.203	0.226	0.240	-0.005	-0.092	0.208	-0.106	0.236	1					
HDL	-0.396	-0.104	-0.435	-0.411	-0.352	-0.235	-0.090	-0.260	0.106	0.295	-0.314	1				
LDL	0.014	-0.041	0.022	0.037	0.037	-0.074	-0.092	0.056	-0.096	**0.893**	0.125	-0.065	1			
RMR	**0.702**	**0.539**	**0.573**	**0.538**	0.287	**0.620**	0.416	0.394	-0.195	-0.016	0.094	-0.241	0.086	1		
VO2Max	0.429	**0.648**	0.183	-0.002	-0.263	**0.710**	0.481	0.314	-0.005	-0.083	-0.019	-0.097	-0.033	0.444	1	
Glucose	0.208	-0.048	0.272	0.279	0.193	0.056	0.167	0.273	0.214	0.057	0.191	-0.082	0.024	0.105	-0.067	1

Correlations greater than |0.5| are in bold, with correlations greater than |0.7| also shaded in gray.

Supplementary Table 2. Results from GWAS Catalog obesity-related genes with RMR

CHR	SNP	Annotation	P-value[a]	Beta[a]	Beta SE[a]	P-value[b]	Beta[b]	Beta SE[b]
22	rs3827354	*PLA2G6*:intron	**0.0002**	3.93E-05	1.07E-05	**0.0018**	3.21E-05	1.03E-05
13	rs2352028	*GPC5*:intron	**0.0032**	3.48E-05	1.18E-05	0.089	1.94E-05	1.14E-05
1	rs1409157	*TBX15*:intron	**0.0037**	-3.21E-05	1.11E-05	**0.0079**	-2.84E-05	1.07E-05
1	rs10493485	*NEGR1*:intron	**0.0039**	4.03E-05	1.39E-05	**0.0052**	3.61E-05	1.29E-05
14	rs368692	*NRXN3*:intron	**0.0044**	-3.18E-05	1.12E-05	**0.0015**	-3.37E-05	1.06E-05
22	rs5756931	*PLA2G6*:intron	**0.0048**	-3.37E-05	1.20E-05	**0.0211**	-2.58E-05	1.12E-05
14	rs10146997	*NRXN3*:intron	**0.0050**	-3.31E-05	1.18E-05	**0.0039**	-3.17E-05	1.10E-05
15	rs17747633	*CASC5*:K1259E	**0.0062**	-3.75E-05	1.37E-05	0.091	-2.23E-05	1.32E-05
10	rs77961654	*TCF7L2*:P477T	**0.0064**	-1.18E-04	4.33E-05	**0.028**	-8.41E-05	3.84E-05
3	rs1801282	*PPARG*:P12A	**0.0065**	5.43E-05	1.99E-05	0.060	3.44E-05	1.83E-05
2	rs1013940	*SLC5A7*:I89V	**0.0065**	5.98E-05	2.20E-05	**0.0024**	6.20E-05	2.04E-05
16	rs117587884	*NLRC5*:D1257N	**0.0072**	1.05E-04	3.90E-05	**0.0099**	9.52E-05	3.69E-05
1	rs984222	*TBX15*:intron	**0.0080**	-3.07E-05	1.16E-05	**0.017**	-2.42E-05	1.02E-05

[a]Model 1 adjusted for age, gender, and principal components. [b]Model 2 additionally adjusted for BMI

Supplementary Table 3. Top Results from single variant association of RMR (not adjusted for BMI) in Children Only

SNP	CHR	POS	Alleles	N	MAF	Beta	Beta SE	PVALUE
rs9561921	13	96293631	G/A	111	0.09	0.000125	2.66E-05	2.52E-06
rs116607188	6	31496949	C/T	111	0.02	-0.00024	5.69E-05	1.65E-05
rs3214631	7	43495878	T/-	111	0.50	6.28E-05	1.50E-05	2.99E-05
rs9908677	17	9712255	A/G	111	0.49	6.11E-05	1.48E-05	3.75E-05
rs148296188	2	232156084	C/T	111	0.01	-0.00031	7.80E-05	7.13E-05
rs13023962	2	1271230	A/G	111	0.35	-6.03E-05	1.54E-05	8.74E-05
rs192180434	5	79032067	G/T	111	0.01	-0.00027	7.02E-05	9.31E-05
rs142926727	5	79035144	C/A	111	0.01	-0.00027	7.02E-05	9.31E-05
rs8016634	14	23282152	G/A	110	0.04	-0.00017	4.26E-05	9.75E-05
rs79573699	17	30348206	C/T	111	0.02	-0.00019	4.98E-05	9.86E-05
